# Prevalence and Characteristics of CAM Use among People Living with HIV and AIDS in Lebanon: Implications for Patient Care

**DOI:** 10.1155/2016/5013132

**Published:** 2016-12-06

**Authors:** Joana Abou-Rizk, Mohamad Alameddine, Farah Naja

**Affiliations:** ^1^Department of Nutrition and Food Sciences, Faculty of Agriculture and Food Sciences, American University of Beirut, Riad El-Solh, Beirut 1107 2020, Lebanon; ^2^Mohammed Bin Rashid University of Medicine and Health Sciences, Building 14, Dubai Healthcare City, P.O. Box 505055, Dubai, UAE; ^3^Department of Health Management and Policy, Faculty of Health Sciences, American University of Beirut, Riad El-Solh, Beirut 1107 2020, Lebanon

## Abstract

This study aimed to assess the prevalence and determinants of Complementary and Alternative Medicine (CAM) use among People Living with HIV and AIDS (PLWHA) in Lebanon and to identify related issues that may affect patient care. A cross-sectional survey design was used to interview 116 PLWHA in Beirut. The questionnaire addressed sociodemographic and disease characteristics as well as CAM use. The main outcome of the study was CAM use since diagnosis. Data analysis included descriptive statistics and logistic regression analyses. Overall, 46.6% of participants reported using one or more CAM therapies, with herbs and herbal products being the most commonly used (63%). A higher education level was associated with a 3-fold increase in the odds of CAM use. Among users, 20% used CAM as alternative to conventional treatment, 48% were not aware of CAM-drug interactions, 89% relied on nonhealth care sources for their choice of CAM, and 44% did not disclose CAM use to their physician. CAM use is prevalent among Lebanese PLWHA. Findings of this study highlighted the need to educate health care practitioners to have an open communication and a patient-centered approach discussing CAM use during routine care and to enhance awareness of PLWHA on safe use of CAM.

## 1. Introduction

Complementary and Alternative Medicine (CAM) refers to a group of diverse medical and health care systems, therapies, and products (e.g., nutritional supplements, herbal remedies, acupuncture, and meditation) that are not presently considered a part of medical training or practice in countries where allopathic medicine forms the basis of the national health care system [1–3]. The use of CAM has been prevalent among many patient populations, especially those with life threatening illness and chronic diseases such as HIV infection and AIDS [4, 5], with the majority using CAM as an adjunct to conventional treatment [6–8]. Reported prevalence estimates of lifetime use of CAM among People Living with HIV and AIDS (PLWHA) reached up to 90% [9], with the most commonly reported forms of CAM used being vitamins and herbs, followed by prayer, meditation, and spiritual healing [7, 9, 10]. Such a high prevalence of CAM use among PLWHA could be due to a variety of reasons, such as the desire to strengthen immunity, improve general wellbeing, and be actively involved in the management of their disease [6, 11–14]. Lessening side effects of Highly Active Anti-Retroviral Therapy (HAART) is another reason why PLWHA resort to CAM [6, 15]. In fact, although the introduction of HAART into clinical practice in 1996 dramatically changed the development of HIV-related diseases [16], it has inflicted a range of side effects, including gastrointestinal and dermatological symptoms, cardiac and liver diseases, and bone loss [9, 17]. In certain low and middle income countries, the limited availability, accessibility, and/or affordability of HAART is considered an additional reason for PLWHA to increasingly seek CAM use [7, 16].

Previous reports have highlighted a potential positive effect of CAM use on quality of life among PLWHA. For instance, a survey of HIV-positive outpatients showed that 70% of participants who used any of the following CAM therapies (exercise, lifestyle changes, dietary supplements, counseling, herbal medications, megavitamins, and prayer therapy) reported an improvement in their quality of life [18]. Furthermore, the results of a randomized prospective controlled trial showed significant differences for quality of life assessment among HIV patients who used massage and stress management compared to controls [19]. Despite the potential beneficial effect that CAM use may have on the quality of life of PLWHA [20, 21], it is important to consider such use in the context of associated risks [7, 16, 21]. For instance, CAM use may interfere with the success of conventional HIV treatment as a result of interactions between ingested forms of CAM with HAART and the possibility that CAM use may impede uptake or adherence to HAART [7, 10–12, 21, 22]. To overcome and reduce these risks, it is recommended that physicians be aware of frequently used CAM therapies, their efficacy, and side effects [9, 12, 15, 23, 24] and where appropriate discuss such use with their patients, in order to improve physician-patient relationship and adherence to HAART and to identify potential safety issues [25, 26]. However the role of the health care provider has been less clear in the context of CAM use, especially with the significant rates of nondisclosure of use reported in the literature [12, 27, 28] and the reliance mainly on family, friends, and the media as main sources of information for the choice of CAM [29, 30]. Hence the assessment of prevalence, predictors, and characteristics of CAM use among PLWHA is important and has critical implications for optimal patient care.

Worldwide, the Middle East and North Africa (MENA) region has the highest increase of new HIV infections (31% since 2001) coupled with the lowest HAART coverage level (11%) [31]. In the MENA region, the risks associated with CAM use are particularly relevant given the barriers to HAART which include stigmatization, lack of medical insurance coverage and infrastructure, interrupted access to HAART, HIV myths, or misconceptions. Furthermore, the use of herbal and alternative therapies is common with the CAM markets being largely unregulated [32–34]. Research characterizing patient behavior and coping mechanisms in the MENA including CAM use has been limited for many reasons, most distinctive of which is the political unrest and conflict frequently experienced by many countries of the region [31]. There has been a dearth of studies characterizing the use of CAM among PLWHA in the region. The objectives of this study are to examine the prevalence and determinants of CAM use among a selected sample of PLWHA and to identify issues which may have implications for patient care such as disclosure of CAM use to the treating physicians and the role of the latter in the patients' choice of CAM.

## 2. Methodology

### 2.1. Study Design and Population

A cross-sectional study assessing the point prevalence, determinants, and characteristics of CAM use among a sample of PLWHA was conducted in Beirut, Lebanon. Ethical approval was obtained from the Institutional Review Board (IRB) for Social and Behavioral studies at the American University of Beirut (AUB) (protocol number NUT.FN.07). Participants were eligible to participate in this study if they were aged 18 years and older with known diagnosis of HIV infection, HIV-related disease, or AIDS. Based on sample size calculations, a sample of 95 patients was needed to estimate CAM use prevalence among PLWHA, at a 95% confidence interval with 5% margin of error, and an assumed prevalence of CAM use of 45%. The latter prevalence was based on previous findings in the literature [6, 11].

The participants were recruited from a large Nongovernmental Organization (NGO) that facilitates the access to medical care and provides moral and social support for PLWHA in Lebanon [35].

### 2.2. Data Collection

Recruitment of PLWHA occurred at the NGO premises during year 2012. During weekly support group meetings, the NGO staff coordinator introduced the study aims and objectives to the attendees. Patients who did not attend these meetings but were registered at the NGO were contacted by phone and were briefed about the study. Interested patients were interviewed by a research assistant in a private room at the NGO premise. An oral consent was obtained from the participants prior the completion of the questionnaire. The written consent was waived to avoid revealing identity of participants. No compensation was offered in order to allow patients to choose voluntarily—without any element of coercion—whether to participate in the study or not. The face-to-face interview approach was chosen for the completion of the questionnaires over self-completion in order to minimize literacy barriers and improve validity of the collected data [36]. Prior to going to the field, the research assistant underwent extensive training to adopt an approachable, motivational, and nonjudgmental attitude in order to achieve higher response rates and minimize data collection related biases.

Patients were reassured that the collected information will not be shared with their health care providers or with the NGO administration. Random identifiers were assigned to participants and completed questionnaires were stored in locked cabinets, with exclusive access to members of the research team.

### 2.3. Survey Instrument

During the interview, patients completed a multicomponent questionnaire, comprised of three sections: the first section included sociodemographic characteristics, such as age, gender, marital status, monthly income, employment status, health insurance, educational level, and crowding index. Crowding index was defined as the average number of people per room, excluding the kitchen and bathroom. Previous studies have shown that a higher crowding index was correlated with a lower socioeconomic status [37, 38]. The second section included disease characteristics, such as the duration since diagnosis with HIV, perceived health status, current use of HAART, CD4 count, and symptoms experienced. The third section of the questionnaire addressed the frequency and types as well as the characteristics of the CAM use, such as the factors influencing CAM choice, reasons for using CAM, rate of disclosure to treating physicians, and CAM-related side effects. CAM use was defined as using CAM at least once after HIV diagnosis and was examined using the following question “Have you used any complementary and alternative therapies/modalities for the treatment of HIV since diagnosis with HIV? If yes, specify:—-?” The questions related to the reasons for using CAM, the side effects of CAM, the source of information on CAM use, and the reasons for not reporting CAM use to a health care provider were all open-ended questions with appropriate probing techniques. Responses were later grouped into the categories reported in the results section. The questionnaire was developed and reviewed by a panel of experts consisting of a nutrition epidemiologist and a health policy expert. The original version of the questionnaire was prepared in English and later was translated to Arabic (since the majority of patients spoke Arabic). A professional translator translated the Arabic version back into English and parallel-form reliability of the questionnaire was examined, whereby the original and the back translated versions were compared for consistency by two bilingual experts. The questionnaire was also pilot tested on a small sample population (*n* = 9) for clarity and cultural sensitivity. During the pilot testing, a few patients inquired about the meaning of certain terms such as “alternate,” “complementary,” and “CAM-drug interactions,” and hence these terms were reworded in the revised questionnaire to enhance clarity. The results of the pilot testing were included in the analysis.

### 2.4. Statistical Analysis

The filled questionnaires were checked for completeness, and responses were coded and entered into the Statistical Package for the Social Sciences (SPSS) software version 23.0 for Windows (SPSSInc., Chicago, IL). Descriptive statistics of participants' sociodemographic, disease, and CAM use characteristics were expressed in frequencies and proportions. Comparisons between CAM users and nonusers characteristics were conducted using chi-square. The association of each of those characteristics with CAM use was assessed using simple logistic regression, with CAM use as outcome variable. In order to evaluate the correlates of CAM use, a multiple logistic regression model was used. In this model, variables were included if they were significantly associated with the outcome in the univariate analysis. Odds ratios and their respective 95% confidence intervals were computed. Statistical significance was detected by a *p* value less than 0.05.

## 3. Results

### 3.1. Prevalence of CAM Use

Over a period of one year, out of 160 patients who were introduced to the study, a total of 116 HIV-infected patients were recruited and completed the study (response rate: 72.5%). When asked by the NGO staff coordinator, patients indicated the following as main reasons for their refusal to participate: fear of personal identification, lack of interest, and lack of time. The sample population consisted of 91 males, 23 females, and 2 transgender adults. The point prevalence of CAM use was 46.5%, 95% CI (37.7–46.5), with 54 patients reporting using a form of CAM since diagnosis with HIV.

### 3.2. Sociodemographic and Disease-Related Correlates

Table 1 displays the sociodemographic and disease characteristics of users and nonusers of CAM among the study participants (PLWHA). Close to two-thirds of study participants were aged 35 years and older (65.5%) with a male majority (78.4%). A considerable proportion of participants had no monthly income (40.9%) and/or were unemployed (47.4%). Almost three-quarters of participants had no social security or insurance coverage (72.4%). In addition, only 49.1% of the study population had a high school or university degree. As for the disease characteristics, 51.7% of the participants have been aware of their HIV status for 6 years or more and 65.5% perceived their health status as good or excellent. A large proportion of the study population was receiving HAART at the time of interview (85.3%), and less than a quarter (23.3%) reported no symptoms. The results of the simple logistic regression analysis showed that, among the factors considered in this study, age, marital status, education, and the crowding index were significantly associated with the use of CAM. The odds of using CAM were lower among participants aged 35 years or more (OR: 0.43, CI: 0.19–0.95). Participants who were married or living with a partner also had lower odds of using CAM as compared to single, separated, or widowed participants (OR: 0.32, CI: 0.14–0.73). A higher education level among participants (high school/university versus less than high school) was associated with a higher odd of CAM use (OR: 4.57, CI: 2.09–10.00). Furthermore, participants reporting a crowding index equal or greater than 2 had a lower odd of using CAM (OR: 0.32, CI: 0.15–0.70) (Table 1).

Multiple logistic regression was used to examine the correlates of CAM use in the study population (Table 2). Variables that were found to be significantly associated with CAM use in the simple logistic regression analysis were included in the multiple regression. Only education level remained significantly associated with CAM use with higher odds observed among participants with a high school or university degree as compared to those with less than a high school diploma (OR: 3.38, CI: 1.48–7.75) (Table 2).

### 3.3. Characteristics and Types of CAM Use

The characteristics and types of CAM use among study subjects (PLWHA) are shown in Table 3. Among CAM users, one in 5 patients (20.4%) used CAM as alternative to HAART. In addition, the most commonly reported reason for using CAM therapies was to improve the general health and ensure long term survival (92.6%). Other reported reasons included the belief that CAM is more natural compared with conventional treatment (55.6%), to improve their nutritional status (22.2%). A small proportion of the CAM users reported using CAM to avoid taking HIV medications (5.6%) and to have more personal control over their health care (3.7%). Close to half of CAM users were not aware of the potential CAM-drug interaction (48.1%). The majority of users would recommend the use of CAM to other HIV patients (74.1%). In addition, most of CAM users relied on personal knowledge, organizations, media, friends, and family for their choice of CAM (66.7%), while 39% reported the treating physician or nurse as their source of information for CAM use. Other reported sources included alternative therapist, traditional healer, or religious leaders (22.2%). Out of the 54 users of CAM, 24 patients (44.4%) did not disclose the use of CAM to their physician. Upon disclosure, the reaction of the physician was rather encouraging (83.3%), with only 13.3% reporting a discouraging reaction (13.3%). The main reason reported for not disclosing CAM use to their physician was the absence of the need of the physician's approval (50.0%). Other reported reasons included fear of lack of understanding of the physician (16.7%), lack of contact with the physician (12.5%), and the certainty that the doctor would not accept the use of CAM (8.3%) (Table 3).

**Figure 1:**
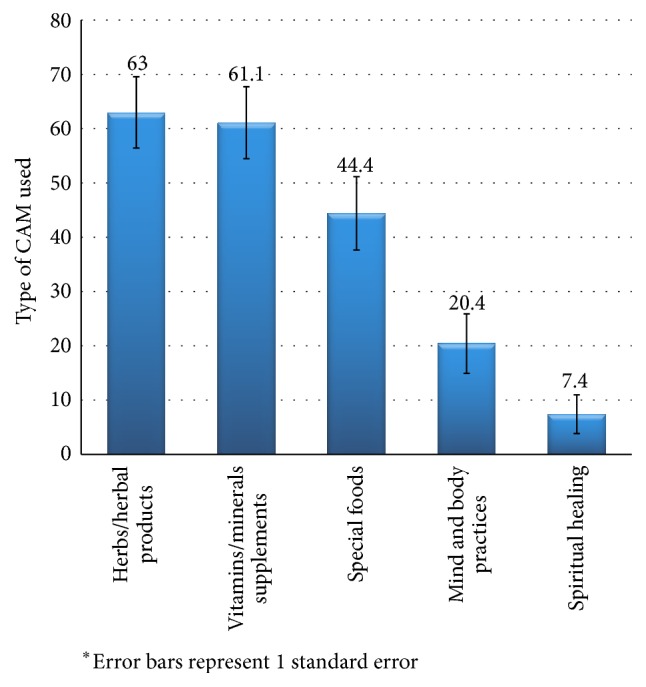
Frequency (%) of the type of CAM used among PLWHA in Lebanon^*∗*^.

Figure 1 illustrates the various types of CAM used by the participants in this study. The most commonly used CAM therapies were found to be herbs and herbal products (63.0%), followed by vitamins and minerals supplements (61.1%). Other types of CAM used included special foods (44.4%), mind and body practices (20.4%), and spiritual healing (7.4%). Herbs and herbal products included the utilization of specific herbs such as baby oak leaves, hibiscus flower, hyssop, rosemary, and nigella sativa seeds and other natural products such as fenugreek oil and grape seeds extract. The most commonly reported vitamins and minerals supplements included multivitamins preparations, vitamin C, vitamin D, and iron supplements. Special foods reported by the participants included probiotics, cranberry juice, grapefruit juice, spinach, and almonds and specific dietary practices. Reported mind and body practices were acupuncture and meditation, while spiritual healing included mainly prayers and spiritual rituals and practices (Figure 1).

### 3.4. Characteristics of Nonusers of CAM

The characteristics of nonusers of CAM among the study participants are shown in Table 4. Among nonusers of CAM, 57.4% stated they would consider using CAM in the future, and close to half of the patients were not aware of drug interaction with CAM. The most commonly reported reasons for not using CAM were the fact that the doctor did not prescribe it (32.3%) and not believe in it (30.6%) (Table 4).

## 4. Discussion

This study aimed to investigate the prevalence and characteristics of CAM use among PLWHA in Lebanon and to examine the implications of this use on patient care. The findings of this study showed that 46% of surveyed patients have used one type of CAM since diagnosis with HIV/AIDS, with the most common CAM therapies used being herbs/herbal products, followed by vitamins and minerals supplements, special foods, mind body practices, and spiritual healing. Among sociodemographic and disease characteristics, a higher education level was positively associated with CAM use. Findings related to CAM use characteristics that may jeopardize patients' health and care were using CAM as alternative to conventional treatment (20%), lack of awareness of CAM-drug potential interaction (48%), and overwhelming reliance on nonhealth care sources for the choice of the CAM therapy to use (89%), as well as lack of disclosure of CAM use to treating physician (44%).

The prevalence estimate of CAM use found in this study is comparable to reports among other HIV study populations. For instance, in Australia, of 151 patients attending HIV clinics, 49% reported using CAM [6]. Similarly, the prevalence of CAM use was found to be 47% in a sample of 682 HIV patients in Vancouver, Canada [11]. A much higher prevalence was reported in Ontario, with 77% of patients using CAM [15]. On the other hand, a review of CAM use studies among PLWHA provided a range of prevalence estimates between 55 and 60% [9]. These observed variations in CAM use between studies could be in part attributed to differences in sociocultural understanding of CAM use and to inequalities in the availability and access to HAART. In addition, methodological differences in study designs and definitions of CAM might have also contributed to the varying prevalence estimates of CAM use by different HIV patients populations [39]. The prevalence estimate in this study was higher compared to the national estimate of CAM use in Lebanon (30%) [33]. This is in line with previous studies showing that patients with chronic diseases are more likely to resort to CAM use as compared to the general population [40, 41].

Among CAM modalities used in this study population, herbs and herbal products were the most common (63%), followed by vitamins and minerals supplementation. These findings are in accordance with a review of CAM therapies used by HIV patients, whereby herbs/herbal products and vitamins and minerals supplementations emerged as the most common approaches used, followed by prayer and spiritual healing [9]. While in this study, the frequency of vitamins and minerals use came second to herbs and herbal products, previous studies conducted in Australia, British Columbia, and Ontario, Canada, reported vitamins and minerals supplementation as the most common CAM used [6, 11, 15]. The prevalent use of herbs and herbal product in this study reflects the rich cultural heritage of herbal medicine in Lebanon and the region. In fact, around 200–250 plant species are still in use in Arab traditional medicine for the treatment of various diseases [42]. Arab families often include in their repertoire of medicinal use many of these plants even though very few have had their medicinal properties investigated using contemporary evidence-based medicine [43, 44]. Furthermore, the general belief that herbal remedies are “natural” and do not cause side effects makes them an attractive method of self-management of disease and provides some sense of control over own health and wellbeing [45].

Consistent with the findings of most studies addressing correlates of CAM use among PLWHA, in this study a higher education level was significantly associated with CAM use [15, 46–50]. Suggested reasons to explain this association include higher levels of health literacy and access to information related to CAM, potential for self-determination, and greater disposable income to spend on health care [51].

Similar to other studies investigating modalities of CAM use among PLWHA, our findings showed that one in five patients used CAM as alternative to conventional treatment [27, 52]. From a patient care perspective, this could cause delays in the start of conventional HIV/AIDS treatments, viral rebound, immune decompensation, clinical progression, and decreased survival time [53–55]. Therefore, although the proportion of PLWHA reporting using CAM as an alternative to therapy is relatively low, the repercussions are quite serious with potential negative consequences on patients, their families, and the health system at large (increased cost of treatment). Factors reported to influence the decision to decline conventional treatment after diagnosis in favor of CAM use included poor doctor-patient communication, the emotional effect of the diagnosis, perceived severity of conventional treatment side effects, a high need for decision-making control, and strong beliefs in holistic healing and the mind-body-spirit connection [56].

Another critical finding in this study is the prevalent lack of awareness of CAM-drug potential interaction (48% among users and 50% among nonusers). This finding is disconcerting especially in light of the evidence that CAM therapies may jeopardize the efficacy of HAART and also contribute to antiretroviral drug resistance. For example, there is some evidence that of an association of Echinacea with increased HIV viral load [57] and of Kava with hepatotoxicity [58]. Furthermore, garlic [59], vitamin C [60], and St John's wort [61–63] may decrease the efficacy of HAART. Aloe may reduce HAART drug absorption, and Ginkgo, Ginseng, and milk thistle may intensify HAART-related side effects [52]. It, hence, becomes imperative for health care practitioners to educate patients on the potential side effects of using CAM in general and using it as an alternative to conventional treatment in particular. The responsibility here is shared between the Ministry of Health as the regulator and funder, the NGOs as the venue where such an education could take place, and the treating physicians who are monitoring the treatment and general health conditions of their patients.

Another point of concern in this study is the high rate of nondisclosure of CAM use to the treating physician. The rate of nondisclosure obtained in this study is comparable to the results of previous reports in the literature which showed that a significant proportion of PLWHA are reluctant to discuss their use of CAM with the physician (53% in Ontario, Canada [15]; 68% in Malaysia [27]). Potential reasons for not discussing the CAM use with the physician could be related to physicians not asking about CAM use or to concerns about physician knowledge regarding CAM rather than to physician discouragement or negativity about the use of CAM [64]. Additional reasons may include patients' concerns for disproval and loss of medical care privileges, in addition to the belief that CAM is safe, holistic, natural, and nontoxic in contrast to conventional medicine, which is usually perceived as depersonalized and not completely effective [65].

The marginal role of the treating physicians in CAM use of PLWHA found in this study is further underscored by the fact that the majority of patients (89%) have relied mainly on nonhealth care related sources. This finding is congruent with previous studies showing that the main sources of information for CAM were personal knowledge, media, friends, and family and not the health care provider [27, 66]. Hence, disclosure and open communication about CAM use with the treating physician is an important part of HIV/AIDs care as it may protect PLWHA from dangerous and unproven therapies as well as maximize the potential health benefits of CAM. To overcome and reduce these risks, it is recommended that physicians be aware of frequently used CAM therapies [9, 12, 15, 23, 24] and where appropriate discuss its use with their patients, in order to improve physician-patient relationship and adherence to HAART and to identify potential safety issues [25, 26].

From a policy and practice perspective a two-pronged strategy could help rectify the situation in Lebanon. Firstly, physicians need to be educated on both the proper use of CAM in PLWHA, as well as the proper communication approach that would encourage patients to disclose use. The role of syndicates of physicians is central in planning, delivering, monitoring, and evaluating such training sessions to physicians in general and those treating PLWHA in particular. The second approach entails revising clinical practice guidelines to integrate investigating the potential use of CAM therapies in the care process of PLWHA. The revision process could be carried out through the joint efforts of the Ministry of Public Health, the syndicate of physicians and educational institutions.

The findings of this study ought to be considered in light of a few limitations. Recruitment of PLWHA is challenging given the stigmatization, social isolation, and fear of identity disclosure. In the context of the study, the most inclusive site of recruitment would have been the Ministry of Health, where PLWHA come routinely to receive their treatment free of charge. However, for ethical consideration and in order to avoid coercion, recruitment of study participants took place in an NGO, where patients come freely and without expectations. Despite the fact that recruited patients come from various areas in Lebanon, the findings of this study may not be generalizable to all PLWHA in Lebanon. Furthermore, patients who are already using a form of CAM may have been more likely to consent for participation in this study than those who were not, hence the potential for a selection bias [67]. However, the 1964 Helsinki Declaration stipulated that, within the consent form, “research subjects must be informed fully about the purpose and methods…”; accordingly such a potential bias could not have been avoided [68]. In addition, the interviewer-based approach in data collection could have incurred a social desirability bias; however the interviewer underwent intensive training to maintain a nonjudgmental and neutral attitude and use standardized techniques and avoiding questions that could influence the subject's responses [36]. Lastly, despite a clear explanation of the purpose of this study and the assurances that participation would not affect eligibility for care or support, interviewed patients may have over exaggerated their financial need in anticipation for additional support; they may have also not fully disclosed their use patterns in fear of loss of benefits.

## 5. Conclusion

This is the first study in the MENA region and in Lebanon to investigate CAM use among PLWHA. Findings of this study showed a prevalent CAM use among Lebanese PLWHA, with education positively associated with CAM use. Among factors implicated in patient health and care were the considerable proportion of patients who used CAM as alternative to conventional treatment, the lack of awareness related to CAM-drug interactions, and the marginal role of health care practitioners in patient's CAM use. The latter was inferred through the findings that the majority of subjects relied on nonhealth care sources for their choice of the CAM therapy and almost half did not disclose the CAM they use to their treating physician. The findings of this study call for a concerted effort by multiple stakeholders in order to enhance the education of both practitioners and PLWHA on the proper and safe use of CAM therapies in complementarity with conventional treatment modalities.

## Figures and Tables

**Table 1:** Sociodemographic and disease characteristics of users and nonusers of CAM among study participants (PLWHA) (*n* = 116)^†^.

	*n* (%) (*n* = 116)	Users of CAM *n* (%) (*n* = 54)	Nonusers of CAM *n* (%) (*n* = 62)	OR (CI)^*∗*^
*Sociodemographic characteristics*				
Age (years)				
<35	40 (34.5)	24 (44.4)	16 (25.8)	1 (ref)
**≥**35	76 (65.5)	30 (55.6)	46 (74.2)	**0.43 (0.19**–**0.95)**
Gender				
Male	91 (78.4)	44 (81.5)	47 (75.8)	1 (ref)
Female	23 (19.8)	9 (16.7)	14 (22.6)	0.69 (0.27–1.74)
Transgender	2 (1.7)	1 (1.9)	1 (1.6)	1.07 (0.06–17.60)
Marital status				
Single/separated/widowed	75 (64.7)	42 (77.8)	33 (53.2)	1 (ref)
Married/living with a partner	41 (35.3)	12 (22.2)	29 (46.8)	**0.32 (0.14**–**0.73)**
Monthly income (USD)				
No income	47 (40.9)	22 (40.7)	25 (41.0)	1 (ref)
<500	21 (18.3)	9 (16.7)	12 (19.7)	0.85 (0.30–2.40)
≥500	47 (40.9)	23 (42.6)	24 (39.3)	1.09 (0.48–2.45)
Employment status				
Unemployed	55 (47.4)	27 (50.0)	28 (45.2)	1.21 (0.58–2.52)
Employed	61 (52.6)	27 (50.0)	34 (54.8)	1 (ref)
Health insurance				
Uninsured	84 (72.4)	37 (68.5)	15 (24.2)	1 (ref)
Insured	32 (27.6)	17 (31.5)	47 (75.8)	1.44 (0.64–3.23)
Educational level				
Less than high school diploma	59 (50.9)	17 (31.5)	42 (67.7)	1 (ref)
High school/university degree	57 (49.1)	37 (68.5)	20 (32.3)	**4.57 (2.09**–**10.00)**
Crowding index^a^				
<2	65 (56.0)	38 (70.4)	27 (43.5)	1 (ref)
≥2	51 (44.0)	16 (29.6)	35 (56.5)	**0.32 (0.15**–**0.70)**

*Disease characteristics*				
Duration of awareness of HIV status				
<6 years	56 (48.3)	27 (50.0)	29 (46.8)	1 (ref)
≥6 years	60 (51.7)	27 (50.0)	33 (53.2)	0.88 (0.42–1.82)
Perceived health status				
Very poor/poor	10 (8.6)	6 (11.1)	4 (6.5)	1 (ref)
Fair	30 (25.9)	11 (20.4)	19 (30.6)	0.39 (0.89–1.67)
Good/excellent	76 (65.5)	37 (68.5)	39 (62.9)	0.63 (0.16–2.42)
Currently receiving HAART				
Yes	99 (85.3)	44 (81.5)	55 (88.7)	0.56 (0.19–1.59)
No	17 (14.7)	10 (18.5)	7 (11.3)	1 (ref)
CD4 count				
<200	10 (11.6)	3 (7.3)	7 (15.6)	1 (ref)
≥200	76 (88.4)	38 (92.7)	38 (84.4)	2.33 (0.56–9.70)
Total number of symptoms reported^b^				
0	27 (23.3)	13 (24.1)	14 (22.6)	1 (ref)
1-2	46 (39.7)	20 (37.0)	26 (41.9)	0.83 (0.32–2.15)
≥3	43 (37.1)	21 (37.1)	22 (35.5)	1.03 (0.39–2.69)

^†^Column total may be different because of missing data.

^*∗*^OR and their 95% CI were derived using a univariate logistic model with CAM use as the dependent variable.

^a^Crowding index was defined as the average number of people per room, excluding the kitchen and bathroom.

^b^The symptoms reported include fatigue, loss of appetite/smell/taste, respiratory, cutaneous, infectious, digestive, orthopedic, and cardiovascular symptoms.

**Table 2:** Correlates of CAM use using multiple logistic regression (OR estimates and 95% CI) among study participants (*n* = 116).

	OR (95% CI)
*Age*	
<35	1 (ref)
**≥**35	0.64 (0.27–1.52)
*Marital status*	
Single/separated/widowed	1 (ref)
Married/living with a partner	0.54 (0.21–1.35)
*Educational level*	
Less than high school diploma	1 (ref)
High school/university degree	**3.38 (1.48**–**7.75)**
*Crowding index* ^a^	
<2	1 (ref)
≥2	0.55 (0.23–1.33)

^a^Crowding index was defined as the average number of people per room, excluding the kitchen and bathroom.

**Table 3:** Characteristics of Complementary and Alternative Medicine (CAM) use in the study population^†^.

	Overall *n* (%) (*n* = 54)
*Characteristics of CAM use*	
Using CAM as alternative or as complementary treatment	
Complementary	43 (79.6)
Alternative	11 (20.4)
Reasons for using CAM^a^	
Improves your general health and ensures long-term survival	50 (92.6)
It is more natural	30 (55.6)
To improve nutritional status	12 (22.2)
It has anti-HIV properties	10 (18.5)
Reduce side effects of conventional medication	9 (16.7)
Family tradition/culture/religious beliefs	6 (11.1)
To avoid taking HIV medications	3 (5.6)
More personal control over your health care	2 (3.7)
CAM monthly expenses (USD)	
<10	20 (37.0)
11–30	18 (33.3)
>30	16 (29.6)
Side effects of CAM^a^	
Digestive symptoms	6 (11.1)
Body, bone, muscle, and/or joints pain	2 (3.7)
Infection symptoms	1 (1.9)
Nutritional status (e.g., weight gain)	1 (1.9)
Believe CAM cures HIV/AIDS^b^	
No/I do not know	30 (55.6)
Yes	9 (16.7)
Expected positive change after the use of CAM^b^	
Maybe	21 (38.9)
Definitely	26 (48.1)
Awareness of drug interactions with CAM	
No/I do not know	26 (48.1)
Yes	28 (51.9)
Would advise other patients to use CAM^b^	
No	6 (11.1)
Yes	40 (74.1)

*Role of treating physician or nurse*	
Source of information on CAM use^a^	
Personal knowledge/media/friends/family/organizations	36 (66.7)
Treating physician or nurse	21 (38.9)
Alternative therapist/traditional healer/religious leaders	12 (22.2)
Reporting CAM use to a Health Care Professional	
No	24 (44.4)
Yes	30 (55.6)
Professional's reaction	
Encouraging	25 (83.3)
Discouraging	4 (13.3)
Neutral	1 (3.3)
Reasons for not reporting to a Health Care Professional^a^ (n = 24)	
Does not need the doctor's approval	12 (50.0)
Fear of not understanding	4 (16.7)
Patient was not in contact with the doctor	3 (12.5)
Doctor will not accept	2 (8.3)

^†^Column total may be different because of missing data.

^a^The values do not sum up to 100% since multiple answer choices could have been selected.

^b^These questions have missing answers.

**Table 4:** Characteristics of nonusers of Complementary and Alternative Medicine (CAM) in the study population (*n* = 6)^†^.

	Overall *n* (%) (*n* = 62)
*Reasons for not using CAM* ^a^	
The doctor didn't prescribe it	20 (32.3)
I do not believe in it	19 (30.6)
I never heard of it	14 (22.6)
Satisfied with conventional treatment	10 (16.1)
Not to have an additional burden	5 (8.1)
Afraid/shy of asking about CAM	3 (4.8)
Unavailability of CAM	3 (4.8)
I am afraid of the adverse effects	2 (3.2)
*If you have not used CAM before, would you consider using CAM?*	
No	26 (42.6)
Yes	35 (57.4)
*Awareness of drug interactions with CAM*	
No/I do not know	32 (51.6)
Yes	30 (48.4)
*Believe CAM cures HIV/AIDS*	
No/I do not know	35 (67.3)
Yes	17 (32.7)

^†^Column total may be different because of missing data.

^a^The values do not sum up to 100% since multiple answer choices could have been selected.

## Abbreviations

AUB: American University of Beirut
CAM: Complementary and Alternative Medicine
HAART: Highly Active Anti-Retroviral Therapy
IRB: Institutional Review Board
MENA: Middle East and North Africa
NGO: Nongovernmental Organization
PLWHA: People Living with HIV and
SPSS: Statistical Package for the Social Sciences.
